# Gastric Band Insertion to Revise Sleeve Gastrectomy: A Case Report

**DOI:** 10.7759/cureus.22999

**Published:** 2022-03-09

**Authors:** Stephanie Pelenyi, Charles K Lee, Orlando Fleites, Frederick Tiesenga

**Affiliations:** 1 Anesthesia, Avalon School of Medicine, Willemstad, CUW; 2 Medical School, Saint James School of Medicine, Park Ridge, USA; 3 General Surgery, West Suburban Medical Center, Chicago, USA

**Keywords:** bariatric medicine, bariatric revision, laparoscopic surgery, adjustable gastric band complications, laparoscopic adjustable gastric band, bariatric surgery

## Abstract

Bariatric surgery for the treatment of obesity, first introduced in the 1950s and 1960s, is now relatively commonplace. Often patients will first have an adjustable gastric band inserted, as this does not require altering or removing parts of the gastrointestinal tract. This procedure is associated with short hospital stays and quick recoveries and may be adjusted without further surgery. Typically only after banding fails mechanically or fails to bring about a satisfactory reduction in body mass index (BMI) do patients undergo further bariatric procedures which involve altering or removing parts of the gastrointestinal tract. Recent research has suggested that gastric banding is associated with greater weight reduction results as a secondary or follow-up procedure following a failed initial bariatric surgery.

Here we report the case of a 43-year-old female with a history of cryptogenic organizing pneumonitis, gastroesophageal reflux disease (GERD), asthma, obesity, and prior sleeve gastrectomy who underwent a laparoscopic gastric band insertion to revise the prior sleeve gastrectomy, in reverse of the typical sequence of bariatric surgeries.

## Introduction

Obesity is a major cause of morbidity and mortality worldwide. While a wide range of treatments are currently implemented for the management of obesity, bariatric procedures, in particular, are recommended as a treatment option for patients with a body mass index (BMI) of 40 kg/m2 (by definition, morbidly/severely/class 3 obese [1]) who instituted but failed a diet and exercise program and presented with obesity-related comorbidities [2,3]. Bariatric surgery is correlated in the literature with longer life expectancy in obese patients when compared to obese patients who do not undergo a bariatric operation [4], and is also associated with remission of diabetes mellitus [5]. Bariatric surgery has also been shown to reduce the risk of gestational diabetes and hypertensive disorders of pregnancy such as pre-eclampsia in women who later become pregnant [6].

Bariatric procedures may be classified into categories, based on the primary mechanism(s) of the procedures on the gastrointestinal (GI) tract. Both sleeve gastrectomy and gastric banding are considered to be restrictive procedures, in which either the size of the stomach is reduced or the stomach’s luminal diameter is reduced through the occupation of space, resulting in satiety with reduced food intake [7]. Gastric banding involves the insertion of a silicone band inflated by saline around the fundus of the stomach; this slows and limits the quantity of food that can be consumed at one time, and weight loss is achieved mainly through this restriction of nutrient intake [8]. Gastric banding is associated with a very low rate of mortality (approximately 0.05%) [9]. Currently, in the United States, only one brand of gastric band retains the approval of the Food and Drug Administration (FDA): Lap-Band (ReShape Lifesciences, San Clemente, CA), which is classified as a weight-loss device [10] and is designed to be inserted laparoscopically. Due to its safety and effectiveness, gastric banding was typically the first choice or initial bariatric operation for surgical treatment of obesity [11]; laparoscopic gastric banding, once estimated to comprise over a third of all bariatric surgeries in the United States a decade ago [12], is now estimated to only account for approximately 1% of all bariatric surgeries in the United States [13].

Sleeve gastrectomy is a procedure in which a large portion of the stomach along the greater curvature is surgically removed. While invasive and permanent, the procedure is associated with dramatic reductions in all-cause mortality among obese adults (59% reduction in mortality in those without type 2 diabetes mellitus, and 30% reduction in mortality in those with type 2 diabetes mellitus) and increased median life expectancy gains compared to non-surgical treatment of obesity (9.3 years in those with type 2 diabetes mellitus and 5.1 years in those without type 2 diabetes mellitus) [14]. Sleeve gastrectomy is now estimated to comprise approximately 60% of all bariatric surgeries in the United States [13].

We present a case wherein a 43-year-old female with an extensive past medical history including cryptogenic organizing pneumonitis, gastroesophageal reflux disease (GERD), asthma, obesity, and prior sleeve gastrectomy underwent a laparoscopic gastric band insertion to revise the prior sleeve gastrectomy, a sequence of bariatric procedures that is in reverse of the typical sequence of bariatric surgeries.

## Case presentation

Our patient is a 43-year-old female presenting with shortness of breath (SOB) and morbid obesity refractory to a sleeve gastrectomy. Previously the patient had undergone a laparoscopic sleeve gastrectomy in 2014; at the time of that operation, she had a BMI of 36 kg/m2. Despite initiating a diet and exercise regimen intended to supplement the procedure and result in weight loss, the patient was unable to lose significant weight. The patient reports SOB with walking a flight of stairs or walking one block.

The patient's past medical history includes asthma (with an exacerbation in 2015), cryptogenic organizing pneumonia (2008), GERD, migraines, and recurrent urinary tract infections (UTIs, 2009 and 2010). In the time between presentation and surgery (two months), the patient was placed on a strict 800-calorie diet, an increased exercise regimen, and had consulted a dietitian.

On examination, the patient was found to have a BMI of 44 kg/m2. The patient was also found to have a cough, shortness of breath, and wheezing. Subsequently, the patient was diagnosed preoperatively with morbid obesity refractory to a prior sleeve gastrectomy. The patient underwent a diagnostic laparoscopy, adhesiolysis, and gastric band insertion. The laparoscopic band was successfully placed without event, and the postoperative diagnosis was morbid obesity refractory to a prior sleeve gastrectomy.

**Figure 1:**
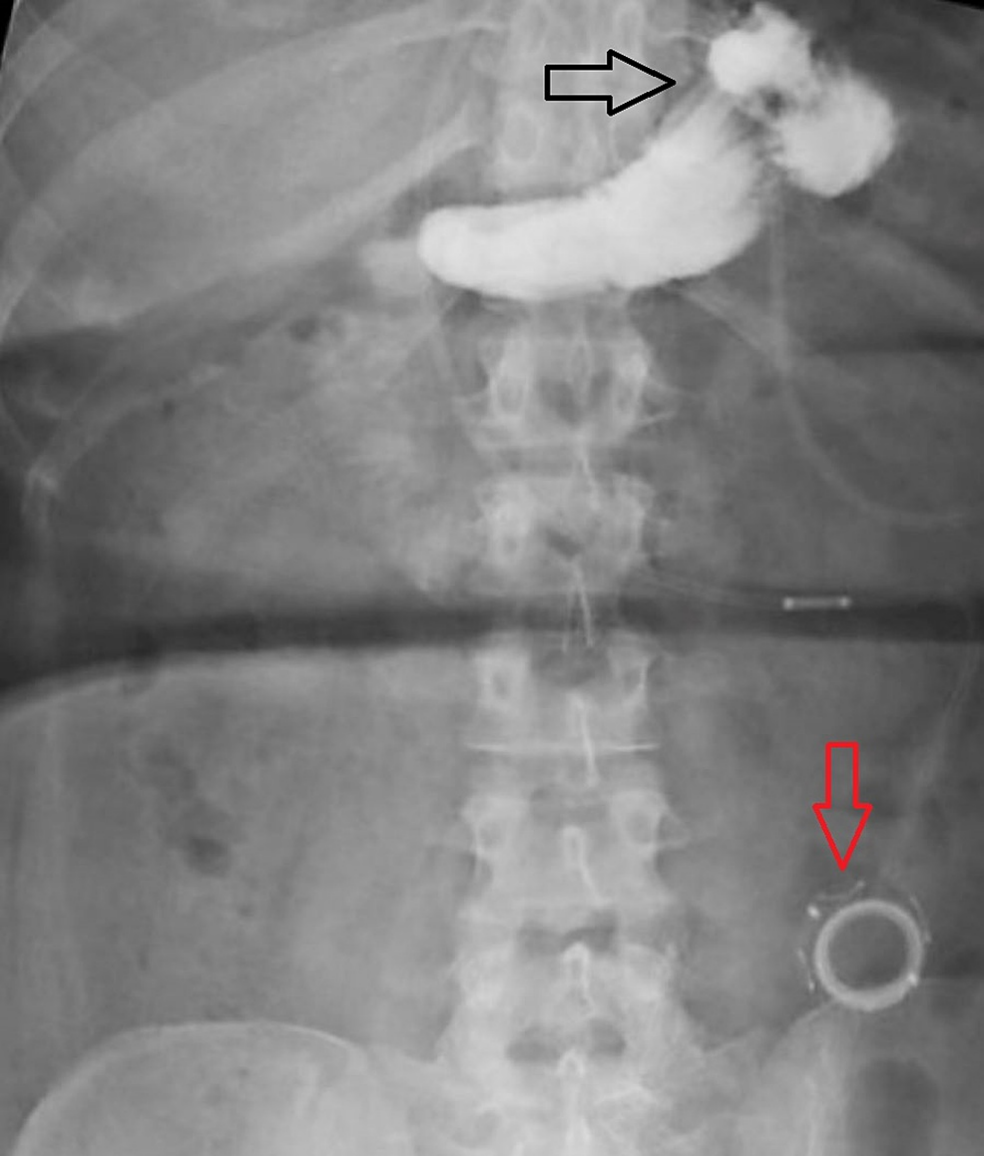
Abdominal radiograph (KUB) showing gastric band (black arrow) and port placement (red arrow).

**Figure 2:**
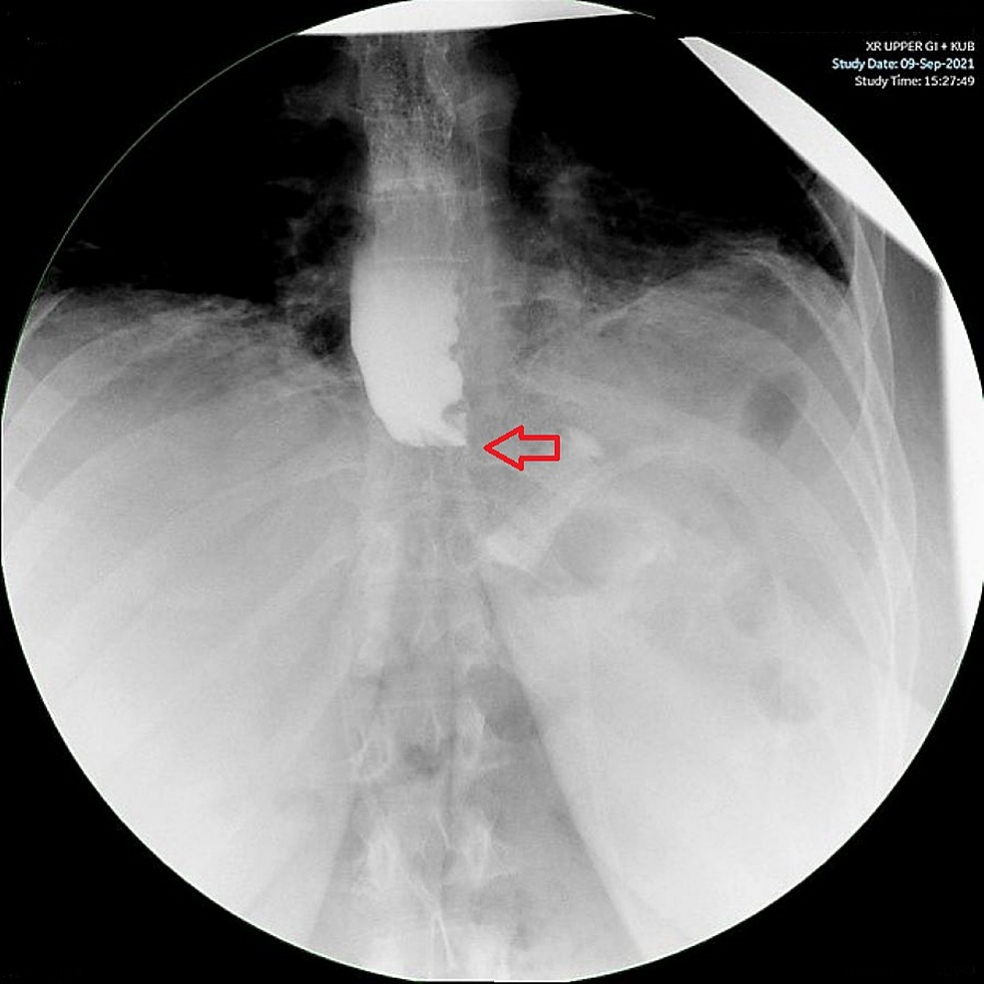
Upper GI radiograph showing retention of contrast, indicating proper function of gastric band and no leakage (red arrow)

The patient’s history of prior bariatric surgery necessitated both an upper GI and an abdominal (or KUB, for “kidney-ureter-bladder”) radiograph, which showed both a properly-placed and functioning gastric band and no evidence of leakage (Figures 1-2). The patient was discharged without event and followed in an outpatient setting. Post-operatively the patient continued to follow up with monthly outpatient visits where she was counseled on diet, exercise, and provided with support groups for regular attendance. During these visits, the gastric band was adjusted as needed. Five months after the insertion of the gastric band, the patient had achieved a BMI of 32 kg/m2, a 27.2% reduction in total BMI from the time of presentation. Using a BMI of 25 as the standard endpoint [15], our patient’s excess BMI loss (% EBMIL) was 63.2%.

## Discussion

Gastric banding was, until recently, the first-choice or initial/primary bariatric operation for surgical treatment of obesity due to its safety and effectiveness [11], as well as the fact that gastric banding does not involve permanent alterations to the GI tract. Until 2011, estimates suggested that over one-third of all bariatric procedures performed in the United States were laparoscopic gastric band insertions [12,13]; the most recent estimates suggest that this has fallen dramatically, such that gastric banding accounts for only 1% of bariatric surgeries, and instead, sleeve gastrectomies now account for some 60% of all bariatric surgeries [13].

Recent research has suggested that the more permanent bariatric procedures such as sleeve gastrectomy or Roux-en-Y gastric bypass, despite the greater risks than banding due to permanent GI tract alterations, should be the initial or first-choice bariatric surgery for treatment of obesity [16-18]. This research is supported by the larger weight reductions associated with the more permanent bariatric surgeries: gastric banding alone has been associated with a 47.5% reduction in weight, gastric bypass alone a 61.6% reduction in weight, gastroplasty (including sleeve gastrectomy) alone a 68.2% reduction in weight, and biliopancreatic diversion/duodenal switch alone a 70.1% reduction in weight [16]. These reports may have contributed to the dramatic increase in sleeve gastrectomies and decrease of gastric band insertions performed over the past decade.

Research reviewing gastric banding as a salvage, secondary, or follow-up procedure to follow the failure of another primary bariatric surgery reported additional reductions in weight and thereby improvements in BMI [19,20], suggesting that the use of gastric banding as a secondary bariatric surgery for obesity is a viable course of treatment. One study found that gastric banding as a salvage procedure to follow-up a failed Roux-en-Y gastric bypass resulted in an additional 20.8% reduction in weight [19], while another found that salvage banding after failed Roux-en-Y gastric bypass resulted in excess BMI loss (% EMBIL) of 55.9%-94.2% [20]. This is consistent with our patient’s outcome, wherein a 27.2% reduction in total BMI (a % EBMIL of 63.2%) was achieved with gastric band insertion as a secondary bariatric surgery after failure of the primary sleeve gastrectomy to achieve reductions in weight and BMI.

## Conclusions

Gastric banding was often the first-choice bariatric procedure performed for the surgical treatment of obesity. However, gastric banding has been replaced over the past decade by sleeve gastrectomy as the preferred bariatric procedure for the surgical treatment of obesity. Despite this, gastric banding as a follow-up or secondary procedure has been shown to be effective for achieving weight reductions in obese patients whose primary bariatric surgery failed to do so. Our case further demonstrates the effectiveness of gastric banding as a secondary bariatric procedure, and suggests that despite gastric banding’s decline in popularity, it should still be strongly considered as an option for obese patients whose primary bariatric surgery failed to achieve satisfactory weight reductions or those patients who are at risk for having a failed primary bariatric surgery.
